# Data-Driven Discrimination, Perceived Fairness, and Consumer Trust–The Perspective of Consumer Attribution

**DOI:** 10.3389/fpsyg.2021.748765

**Published:** 2021-09-30

**Authors:** Luping Sun, Yanfei Tang

**Affiliations:** ^1^Business School, Central University of Finance and Economics, Beijing, China; ^2^Antai College of Economics and Management, Shanghai Jiao Tong University, Shanghai, China

**Keywords:** data-driven discrimination, perceived fairness, consumer trust, attribution, consumer self-concept clarity

## Abstract

With the development of consumer-centric data collection, storage, and analysis technologies, there is growing popularity for firms to use the behavioral data of individual consumers to implement data-driven discrimination strategies. Different from traditional price discrimination, such data-driven discrimination can take more diverse forms and often discriminates particularly against firms’ established customers whom firms know the best. Despite the widespread attention from both the academia and the public, little research examines how consumers react to such discrimination enabled by big data. Based on attribution theory, this paper examines how different ways of consumer attribution of data-driven discrimination influence perceived fairness and consumer trust toward the firm. Specifically, we hypothesize that controllability by consumers and locus of causality of data-driven discrimination interactively influence perceived fairness, which further affects consumer trust. We conduct two experiments to test the hypotheses. Study 1 uses a 2(controllability: high vs. low)×2(locus of causality: internal vs. external) between-subjects design. The results show a significant interaction between controllability and locus of causality on consumer trust. When consumers attribute data-driven discrimination to themselves (internal attribution), consumer trust is significantly lower in low-controllable situations than that in high-controllable situations. When consumers attribute the discrimination to the firm (external attribution), however, the impact of controllability on consumer trust is nonsignificant. Moreover, we show that perceived fairness plays a mediating role in the interaction effect of controllability and locus of causality on consumer trust. Study 2 uses a similar design to replicate the findings of Study 1 and further examines the moderating role of consumer self-concept clarity. The results show that the findings of study 1 apply only to consumers with low self-concept clarity. For consumers with high self-concept clarity, regardless of the locus of causality (internal or external), consumer trust is significantly higher in high-controllable situations than that in low-controllable situations. Finally, we discuss the theoretical and managerial implications and conclude the paper by pointing out future research directions.

## Introduction

Due to the advancement of data storage technologies and big data analytics, increasingly more firms have been tracking and analyzing individual consumers’ online shopping behaviors (e.g., online search, browsing, and purchasing). Based on the insights obtained from such behavioral data, firms are able to implement data-driven discrimination against certain groups of consumers, especially the established or loyal ones (Hannak et al., 2014). Previous research has not clearly defined the concept of data-driven discrimination, but researchers have discussed its similarities to and differences from traditional discrimination (Žliobaitė and Custers, 2016; Criado and Such, 2019).

In essence, data-driven discrimination is one special type of discrimination resulting from the convergence of “big data” and predictive analytic techniques or algorithms. Different from traditional price discrimination, this type of discrimination enabled by advanced data analytics can take more diverse forms and is not limited to the dimension of price. For example, the firm may offer coupons or gifts only to customers who are found to be more sensitive to promotions by algorithms. Second and more importantly, data-driven discrimination may especially discriminate against firms’ established customers whom firms know the best, the practice of which has been known as loyalty penalty (Parker, 2021). Thus, it differs from personalized marketing or loyalty program, which is used by firms to better serve their loyal customers through analyzing customer behavioral data (Stourm et al., 2020). This may be the reason why data-driven discrimination has raised widespread controversy among the public. Finally, compared to traditional price discrimination, data-driven discrimination may not be easily detected by consumers through social comparison. This is because online product or service information is usually delivered privately to each individual customer’s account, which makes it difficult for consumers to compare with others. However, the detection of common forms of data-driven discrimination can still be relatively easy through other ways (e.g., by browsing anonymously rather than using the regular account), especially as consumers in the marketplace have been becoming more and more sophisticated. It is no wonder that increasingly more consumers report being discriminated by firms’ algorithms.

Recently, such data-driven discrimination is prevalent in a broad spectrum of sectors, including but not limited to airline or hotel reservation, online ride-hailing service, and online retailing. According to a survey by China Youth Daily in 2018, 63.4% of the respondents believed that it was common for firms to use data analytics to discriminate, and 51.3% of the respondents had been discriminated in some ways by such data-driven strategies (China Youth Daily, 2018). Despite its prevalence in practice, it remains unclear how consumers would respond to such data-driven discrimination. According to a survey conducted by the Beijing Consumer’s Association in 2019, 83.74% of consumers among the 3,185 interviewees believed that such data-driven discrimination infringed consumers’ right to fair deals, which was perceived to be very unfair. Some other consumers, however, believed that such discrimination is relatively acceptable (Beijing Consumer’s Association, 2019).

Why do consumers respond to such data-driven discrimination differently? As the extremely important equity for a firm (Sirdeshmukh et al., 2002; Mal et al., 2018), whether and when can consumer trust toward the firm be eroded by such data-driven discrimination? This paper intends to answer these questions. Prior research on consumer response to price discrimination mainly examines its general negative effect on perceived fairness and trust. For example, Garbarino and Maxwell (2010) show that price discrimination that violates social norms (e.g., setting prices based on individual consumer demand) is perceived to be more unfair, which further adversely influences consumer trust. Grewal et al. (2004) also suggest that pricing tactics contrary to norms, such as charging a frequent customer more, engender less trust than pricing tactics consistent with norms. Very few studies, however, examine whether and when consumer response to a particular form of discrimination can be more or less negative, especially when the discrimination is data-driven and apparently violates social norms. As being discriminated by algorithms can be interpreted in various ways, consumers may attribute the discrimination differently. For instance, being discriminated may be either because of the firms’ abuse of consumer personal data without their explicit consent or because of loyal customers’ careless disclosure of their personal information to the firm. With different attributions, reactions to such discrimination can vary. Therefore, from a theoretical point of view, consumer response to data-driven discrimination may to a large extent depend on their attribution of the discrimination. Yet, little research examines consumer response to data-driven discrimination from the perspective of attribution.

It is worth noting that some research has examined consumer fairness perception of price increases from the attribution perspective (e.g., Vaidyanathan and Aggarwal, 2003). However, different from simple price increases, price discrimination involves charging different prices to different customers, the practice of which can be more likely to violate social norms and thus arouse more unfair perceptions. More importantly, whether to practice price discrimination is the firm’s free choice and thus is fully controlled by the firm and cannot be justified by uncontrollable factors like a price increase does (e.g., cost surge). Likewise, whether to practice data-driven discrimination is also fully controlled by the firm, and thus, a firm perspective (as in previous research) when making attributions regarding controllability is no longer appropriate. Instead, the controllability by consumers toward data-driven discrimination can be either high or low, depending on whether consumers can avoid being discriminated through changing their profile settings or shopping behaviors. Thus, in our research setting, we innovatively adopt a consumer perspective when examining the attribution of data-driven discrimination.

Specifically, this paper discusses the controllability and locus of causality of data-driven discrimination from a consumer perspective and examines how different attributions influence perceived fairness and consumer trust toward the firm. Based on previous literature, we hypothesize that controllability by consumers and locus of causality of data-driven discrimination may interactively influence perceived fairness, which further affects consumer trust toward the firm. Furthermore, as a boundary condition, we also explore the moderating role of consumer self-concept clarity in the above relationship. These investigations would not only extend theoretical literature on attribution theory and consumer response to discrimination, but also provide important implications for firms practicing data-driven discrimination.

Below, we first develop the research hypotheses and introduce our research framework. Then, we conduct two laboratory experiments to test the hypotheses. Finally, we briefly summarize the findings and implications and conclude the paper with discussing the future research directions.

## Research Hypotheses

### Attribution of Data-Driven Discrimination and Perceived Fairness

Prior research has mainly examined data-driven discrimination in the public sectors from the perspective of social justice. These studies usually focus on the gender, religion, or race biases generated by data-driven discrimination and discuss related legal issues and ethical concerns (e.g., Kim, 2016; Gillis and Spiess, 2019; Kennedy, 2021). In online shopping, data-driven discrimination has been even more common. Online retailers usually discriminate against customers based on their past shopping behaviors rather than innate traits (e.g., race and gender; Ezrachi and Stucke, 2016). For instance, Hannak et al. (2014) examined 16 top e-commerce sites in the United States and found that nine of them were relying on consumer behavioral data to perform price discrimination and price steering. Relatively little academic research, however, examines data-driven discrimination in online shopping and its impact on consumer behavior.

In this paper, we define data-driven discrimination in online shopping narrowly as firms’ strategy or practice to discriminate against customers who are loyal and insensitive to certain marketing stimuli through analyzing customer behavioral data (using algorithms or machine learning methods). With the aid of data analytics, discrimination with loyalty penalty becomes more pervasive and has aroused widespread attention (Addleshaw Goddard LLP, 2018; Parker, 2021). Previous research on price discrimination shows that consumers generally respond negatively to price discrimination that violates social norms (e.g., Garbarino and Maxwell, 2010). However, few studies have examined when consumers can respond more or less negatively to data-driven discrimination, which apparently violates social norms. Faced with different forms of data-driven discrimination, consumer perceptions and reactions may differ. Even in face of the same form of data-driven discrimination, the response of different consumers may also be divergent. This paper examines consumer response to data-driven discrimination in online shopping from the theoretical perspective of attribution.

Weiner’s (1979) attribution theory proposes that people try to make causal inferences about observed behaviors, and these inferences will affect their responses. According to attribution theory, there are three dimensions of attribution: controllability, locus of causality, and stability (Weiner, 1979). Controllability refers to whether consumers believe that the reasons that affect their success or failure can be changed by personal will. When consumers attribute product failure to the firm’s controllable behavior, they will feel angry and intend to hurt the firm (Weiner, 1980; Folkes, 1984). Locus of causality refers to consumers’ belief that their success or failure is caused by individual reasons or by external factors. If consumers think that negative consequences are related to a firm, they may intend to reduce the benefits of the firm. While stability refers to whether consumers believe that the reasons affecting their success or failure are stable, whether they are consistent in similar situations, and whether they will change over time. Researchers have already found that the stability of firm behaviors affects consumers’ expectations of whether future results will change and then affects consumer perceived fairness and trust (Kalapurakal et al., 1991; Nikbin et al., 2012, 2016). Thus, this paper will focus only on the impact of controllability and locus of causality on perceived fairness and consumer trust.

While applying attribution theory to explain consumer behavior, previous research mostly examines how the controllability by the *firm* affects consumer response. When consumers believe that the firm has volitional control over the negative consequences, they are more likely to show negative emotions and responses toward the firm. For example, when consumers find that product failure is caused by the firm’s controllable behaviors, they will be much angrier and may even take actions to hurt the firm (e.g., spreading negative word-of-mouth and purchasing less; Folkes, 1984). In the research setting of data-driven discrimination, the firm is usually believed to have full control over its discrimination strategy based on data analytics. Consumers, however, may or may not be able to exert any influence on whether to be discriminated. According to the dual entitlement principle, both firms and consumers have the right to obtain corresponding benefits (Urbany et al., 1989). As the control of the firm is enhanced and higher profits are obtained, the control of consumers can be weakened, and unfair perceptions may be induced. However, relatively little research examines the impact of the controllability by *consumers* on fairness perception in new digital settings such as data-driven discrimination.

In this paper, we define the controllability of data-driven discrimination from a consumer perspective, which captures whether consumers can avoid being discriminated by changing their own profile settings or behaviors. To achieve discrimination, the firm needs both algorithms and consumer behavioral data. The algorithms are programmed instructions and cannot be controlled by consumers. As the input to algorithms, consumer behavioral data, however, are generated by the consumers themselves and thus can be controlled. Consumers agree the firm to monitor their shopping behavior and in return the firm promises to offer products or services that are tailored to consumers’ personal needs and interests (e.g., more personalized offers or promotions). Realizing that the firm is using their personal data to discriminate against them, consumers may opt out of their agreement with the firm for sharing behavioral data. In practice, they may also strategically adjust their shopping behaviors to avoid being discriminated. For example, consumers can anti-discriminate through deleting or adjusting their cookie settings, disguising themselves as nonacquaintances by browsing anonymously, and putting an interested product into the shopping cart without checking out (CBC News, 2015). Therefore, in the marketplace where consumers have been increasingly sophisticated, the controllability of data-driven discrimination perceived by consumers has becoming higher.

For those who perceive higher controllability, they may have avoided being discriminated through adjusting their own profile settings or shopping behaviors. Thus, these consumers are likely to treat data-driven discrimination as a game in which they can exert some influence toward their own benefits (CBC News, 2015). For example, when consumers are interested in buying an item, they may put it into the shopping cart without checking out and wait until the firm offers a coupon for exactly the same item (i.e., the so-called waiting game). In scenarios like this, consumers can actually benefit from the firm’s practice of data-driven discrimination through strategically altering their shopping behaviors. As a result, their perceived unfairness toward data-driven discrimination may not be very high. For consumers who cannot avoid being discriminated no matter how they change their behaviors (low controllability), they may fully blame the firm for putting them at disadvantage. Thus, consumers may believe that the firm obtains undeserved profits at the expense of their interests. According to the dual entitlement principle, when certain firm practice (e.g., a price increase) increases the firm’s profits beyond its reference or deserved entitlement, consumers will feel more unfair (Kahneman et al., 1986; Novoseltsev and Warlop, 2002). Hence, for consumers with low controllability, the discrimination may not be easily rationalized or justified, which makes consumers perceive data-driven discrimination to be more unfair. Thus, we conjecture that perceived fairness of consumers with high controllability is significantly higher than that of those with low controllability. Hence, we propose H1a.

*H1a*: Perceived fairness toward data-driven discrimination is higher for consumers with high controllability than those with low controllability.

In our research setting, locus of causality refers to whether being discriminated is caused by consumers themselves or by other parties (e.g., the firm). From a consumer perspective, if consumers believe that they are discriminated due to their own reasons (e.g., they accidentally clicked the button of “Agree to Enterprise Access to Personal Data”), it is internal attribution. On the other hand, if consumers believe that being discriminated is caused by other factors (e.g., the firm’s abuse of consumer personal data), then it is external attribution. When consumers make an external attribution, it is highly likely that they consider the firm to be fully responsible for the discrimination. This is because whether to discriminate against certain consumers using algorithms mainly depends on the firm’s free choice of strategy. In this case, consumers may infer that the firm is abusing the collected consumer behavioral data to maximize its own profits at the expense of the consumers’ benefits, and thus, consumer perception of unfairness can be rather strong (Vaidyanathan and Aggarwal, 2003). By contrast, when consumers make an internal attribution, they may mainly blame themselves rather than the firm for being discriminated. Thus, consumers’ unfair perception can be relatively weak. As a result, compared with external attribution, consumer perceived fairness is higher when they internally attribute data-driven discrimination. Therefore, we proposed H1b.

*H1b*: Compared with external attribution, consumer perceived fairness is higher when consumers attribute data-driven discrimination internally to themselves.

Furthermore, controllability and locus of causality may have an interaction effect on perceived fairness. When consumers attribute data-driven discrimination to the firm or other parties (external attribution), they blame the firm or other parties for being discriminated. The unfairness perception is so high that it may lead to strong negative emotions (e.g., anger) among consumers (Pillutla and Murnighan, 1996; Hofman et al., 2012; Claudia, 2013). Consumers who are invoked strong negative emotions may tend to activate System 1 for information processing (Farrell et al., 2014; Laakasuo et al., 2015) and may not cognitively evaluate whether and how the controllability by them will make a difference. In addition, when consumers believe that it is the firm’s fault, they may perceive it highly unacceptable if they need to make efforts to avoid being discriminated (even if they could have done so). Instead, it is the firm that should take responsibility and change its strategy to help consumers avoid such discrimination. In this case, controllability may not matter much for consumers. Therefore, under external attribution, controllability may have a relatively weak impact on fairness perception.

When consumers attribute data-driven discrimination internally to themselves (internal attribution), however, controllability by consumers may make a difference. In this circumstance, consumers mainly blame themselves for being discriminated. Thus, they are less likely to generate very strong negative emotions. In this circumstance, System 2 is relatively more likely to be activated (Farrell et al., 2014). This induces consumers to consider how they could have done to avoid being discriminated. In other words, consumers may cognitively seek ways to get rid of the discrimination, which makes controllability more important. Moreover, when consumers consider themselves as responsible for being discriminated, they may feel more obligated to make efforts to improve the situation they are involved in. In this case, high vs. low controllability can be crucial in the formation of fairness perception. As a result, under internal attribution, controllability may have a much stronger impact on fairness perception.

Based on the above speculation, we propose H1c.

*H1c*: Controllability and locus of causality of data-driven discrimination have an interaction effect on consumer perceived fairness. Compared with external attribution, controllability has a stronger positive impact on consumer perceived fairness under internal attribution.

### Attribution of Data-Driven Discrimination and Consumer Trust

Consumer trust plays an important role in managing customer relationship and maintaining the long-term development of a firm (Kennedy et al., 2001; Andersen and Kumar, 2006). Recent research suggests that consumer perceived fairness is positively correlated with consumer trust (Hubbell and Chory-Assad, 2005; Lee, 2018). For instance, Mushagalusa et al. (2021) examine the impact of price fairness in financial institutions and show that price fairness positively affects consumer trust and satisfaction, thereby reducing customer switching intentions. Therefore, we conjecture that the impact of controllability and locus of causality on consumer perceived fairness may be transferred to consumer trust.

Specifically, compared to low controllability, consumers with high controllability may believe that the firm still offers some opportunities for them to avoid being discriminated. These consumers may perceive the firm to be relatively more conscientious than firms that do not leave any way for consumers to anti-discriminate (i.e., low controllability). Thus, these consumers may have higher trust toward the firm than those with low controllability. Hence, we propose H2a.

*H2a*: The higher controllability of data-driven discrimination by consumers, the higher consumer trust.

Regarding locus of causality, when consumers attribute the discrimination to themselves, they tend to focus on how to make a difference and are relatively more likely to forgive the firm for practicing data-driven discrimination. By contrast, for consumers who attribute the discrimination to other parties, especially the firm, they are more likely to form very strong negative emotions toward the firm. In this case, they are less likely to trust the firm anymore. Hence, we propose H2b.

*H2b*: Compared with external attribution, consumer trust is higher when consumers internally attribute the data-driven discrimination.

In a similar vein, we propose that there is an interaction effect between controllability and locus of causality on consumer trust (H2c). Compared to consumers under internal attribution, consumers who attribute the discrimination externally to the firm or other parties tend to experience stronger negative emotions toward the firm (e.g., anger). This may erode consumer trust toward the firm irrespective of how controllable the discrimination is by consumers. Thus, we conjecture that the positive impact of controllability on consumer trust is stronger for consumers with internal attribution than those with external attribution. Hence, we propose H2c.

*H2c*: Controllability and locus of causality of data-driven discrimination have an interaction effect on consumer trust. Compared with external attribution, controllability has a stronger positive impact on consumer trust under internal attribution.

Substantial research shows that trust is gradually generated based on a series of favorable (or fair) interactions with other people or things (Holmes, 1991; Holtz, 2013). If consumers believe that a firm is fair, then the firm is also perceived to be more credible (Van den Bos et al., 1997). Fairness perception is found to be an effective predictor of individual’s trust in an organization (Hubbell and Chory-Assad, 2005). Furthermore, previous research shows that perception of fairness significantly increases consumer satisfaction and trust (Mushagalusa et al., 2021). Thus, the dominant research on trust and fairness indicates that trust is a result of fairness. Thus, based on H1c and H2c, we further propose that the interaction between controllability and locus of causality affects consumer trust through perceived fairness (H3).

*H3*: Perceived fairness mediates the interaction effect of controllability and locus of causality of data-driven discrimination on consumer trust.

### The Moderating Role of Consumer Self-Concept Clarity

Self-Concept Clarity refers to the degree to which individuals define their self-concept clearly and accurately (Campbell et al., 1996). Individuals with high self-concept clarity have more consistent self-belief and are less likely to change the description of their own personality traits (Campbell, 1990). Related research suggests that consumers with low self-concept clarity are more likely to be affected by marketing strategies (Mittal, 2015) and are more likely to purchase products recommended by sales assistants (Lee et al., 2010). For these consumers, in face of data-driven discrimination, their trust toward the firm may also be more easily swayed by other factors such as the locus of causality and controllability perceived by consumers. When attributing data-driven discrimination externally to the firm, consumer evaluation of the firm can be very low, which may lead to a negative halo effect toward the firm (Beckwith and Lehmann, 1976; Boatwright et al., 2008). In this case, regardless of high vs. low controllability, consumer trust toward the firm can be very low. Under internal attribution, however, consumers mainly blame themselves for being discriminated, and thus, they may be more likely to take actions to change the present situation. In this case, controllability of data-driven discrimination becomes more important, and it may have a much greater impact on consumer trust.

By contrast, consumers with high self-concept clarity mainly make decisions based on their independent opinions (Setterlund and Niedenthal, 1993). Previous research suggests that these consumers are more problem-solving-oriented in decision-making (Bechtoldt et al., 2010). Thus, when being discriminated, these consumers care more about how to change the present situation and get rid of discrimination through proactive behaviors, irrespective of internal vs. external attribution. In this case, high vs. low controllability perceived by consumers can be crucial for them to form attitude toward the firm. Specifically, high controllability would lead to much higher consumer trust than low controllability. That is to say, controllability may generally have a significant positive effect on consumer trust no matter whether consumers internally or externally attribute the data-driven discrimination.

As a result, we conjecture that consumer self-concept clarity moderates the interaction effect between controllability and locus of causality on consumer trust, and propose H4a and H4b.

H4a: For consumers with low self-concept clarity, controllability and locus of causality of data-driven discrimination have an interaction effect on consumer trust. Compared with external attribution, controllability has a stronger positive impact on consumer trust under internal attribution.

H4b: For consumers with high self-concept clarity, controllability and locus of causality of data-driven discrimination have no interaction effect on consumer trust. Compared with low controllability, high controllability significantly improves consumer trust, no matter whether it is external attribution or internal attribution.

**Figure 1:**
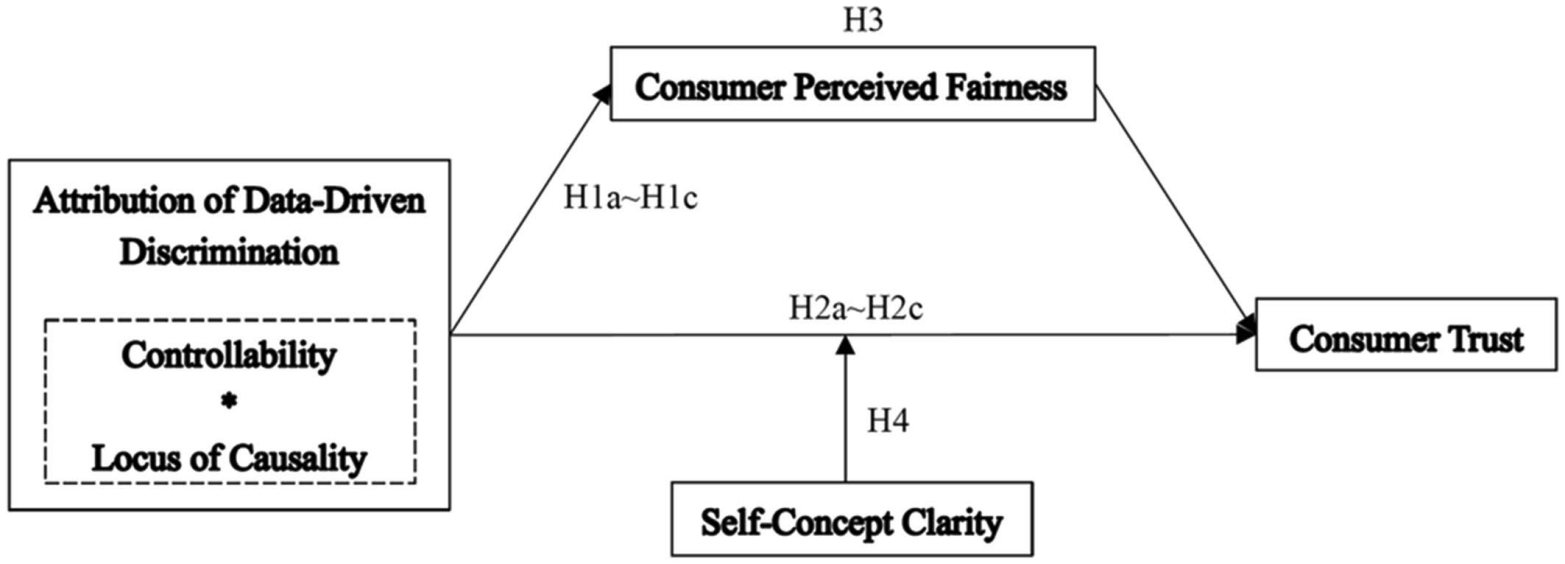
Research Framework.

Our research framework is shown in Figure 1. In the next section, we will test our theoretical framework using two lab experiments.

## Study 1: the Impact of the Attribution of Data-Driven Discrimination

### Method

Study 1 is a 2(controllability: high vs. low)×2(locus of causality: internal vs. external) between-subjects design. We conducted the experiment on one of the largest data collection platforms in China. A total of 137 qualified undergraduate students participated in the experiment and were randomly assigned into one of the four conditions.

During the experiment, we first asked the subjects to imagine that they were discriminated by being imposed an additional service fee (compared to other consumers) while booking tickets online. The booking website took advantage of their database to analyze the behavior of different consumers and intentionally chose certain “prospective” loyal customers to charge them “Gold Medal Service” fees as a default option. All subjects read the same description of such a data-driven discrimination scenario. The only difference for the four conditions is the manipulation of consumer attribution of the website’s discrimination.

For internal attribution conditions, the subjects imagined that they were discriminated because they accidentally clicked the button of “Agree to Enterprise Access to Personal Data” when they registered at the booking website. For external attribution conditions, the subjects imagined that they were discriminated because the online booking website (similar to other booking websites) tracks and uses consumers’ behavioral data without their explicit consent. To manipulate low controllability, we asked the subjects to imagine that they could not change their status of being discriminated at the booking website even if they change their profile settings or browsing behaviors. In high-controllable conditions, however, the subjects imagined that they could change the status of being discriminated at the booking website by changing their profile settings or browsing behaviors (e.g., frequently visiting discount tickets).

After the subjects read the scenario, they reported their perceived fairness regarding the discrimination and their trust toward the booking website (on 5-point scales). We also asked the subjects to report how much they feel angry, frustrated, guilt, and anxious when facing with the scenario. This is because previous research suggests that emotion plays a non-negligible role in the formation of fairness judgments and subsequent fairness-related decision-making (Barsky et al., 2011; Zheng et al., 2017; Barclay and Kiefer, 2019). For example, Campbell (2007) suggests that emotional reactions affect perceived price fairness. Pillutla and Murnighan (1996) indicates that when respondents perceive ultimatum offers unfair, they will feel angry and reject the offer. Existing research has also found that disadvantaged-unfairness is stronger than advantaged-unfairness and that the disadvantaged-unfairness will cause negative reactions such as negative emotions, reduced demand, and the spread of negative word-of-mouth (Martins, 1995; Xia et al., 2004).

As a manipulation check for controllability toward the discrimination, the subjects indicated how much they agree with the following statements: “I can strategically change my behavior to avoid being discriminated,” “No matter how I change my behavior, I cannot avoid being discriminated,” and “The situation of being discriminated is something that I can control by strategically changing my behavior” (5-point scale, 1=strongly disagree; 5=strongly agree). Regarding the manipulation check for the locus of causality, we asked the subjects to indicate their agreement with the following statements: “I am discriminated due to my own reasons,” and “I am discriminated due to other reasons instead of myself” (5-point scale, 1=strongly disagree; 5=strongly agree). Finally, the subjects left their demographic information (e.g., gender and age) and were compensated with a cash lottery.

### Results

#### Manipulation Check

The results show that the manipulation of attribution is successful. Specifically, the evaluation of the controllability in high-controllable conditions is significantly higher than that in low-controllable conditions (*M*_high controllability_=3.47, *M*_low controllability_=2.55, *F*(1,135)=33.967, *p*<0.001). Compared with external attribution conditions, the subjects in the internal attribution conditions are significantly more inclined to believe that they are discriminated by the website because of their own reasons (*M*_internal attribution_=2.61, *M*_external attribution_=1.98, *F*(1,135)=17.247, *p*<0.001).

#### Perceived Fairness

A linear regression model with controllability (0=low controllability, 1=high controllability) and locus of causality (0=external attribution, 1=internal attribution) as independent variables and perceived fairness as the dependent variable was conducted. The results show that the effect of controllability is significant. Compared with the low-controllable conditions, perceived fairness is significantly higher in high-controllable conditions (*M*_high controllability_=2.25, *M*_low controllability_=1.83, *t*(134)=2.277, *p*<0.05). Therefore, H1a is supported. The effect of the locus of causality on perceived fairness, however, is not significant (*t*(134)=0.624, *p*=0.534). Thus, H1b is not supported.

**Figure 2:**
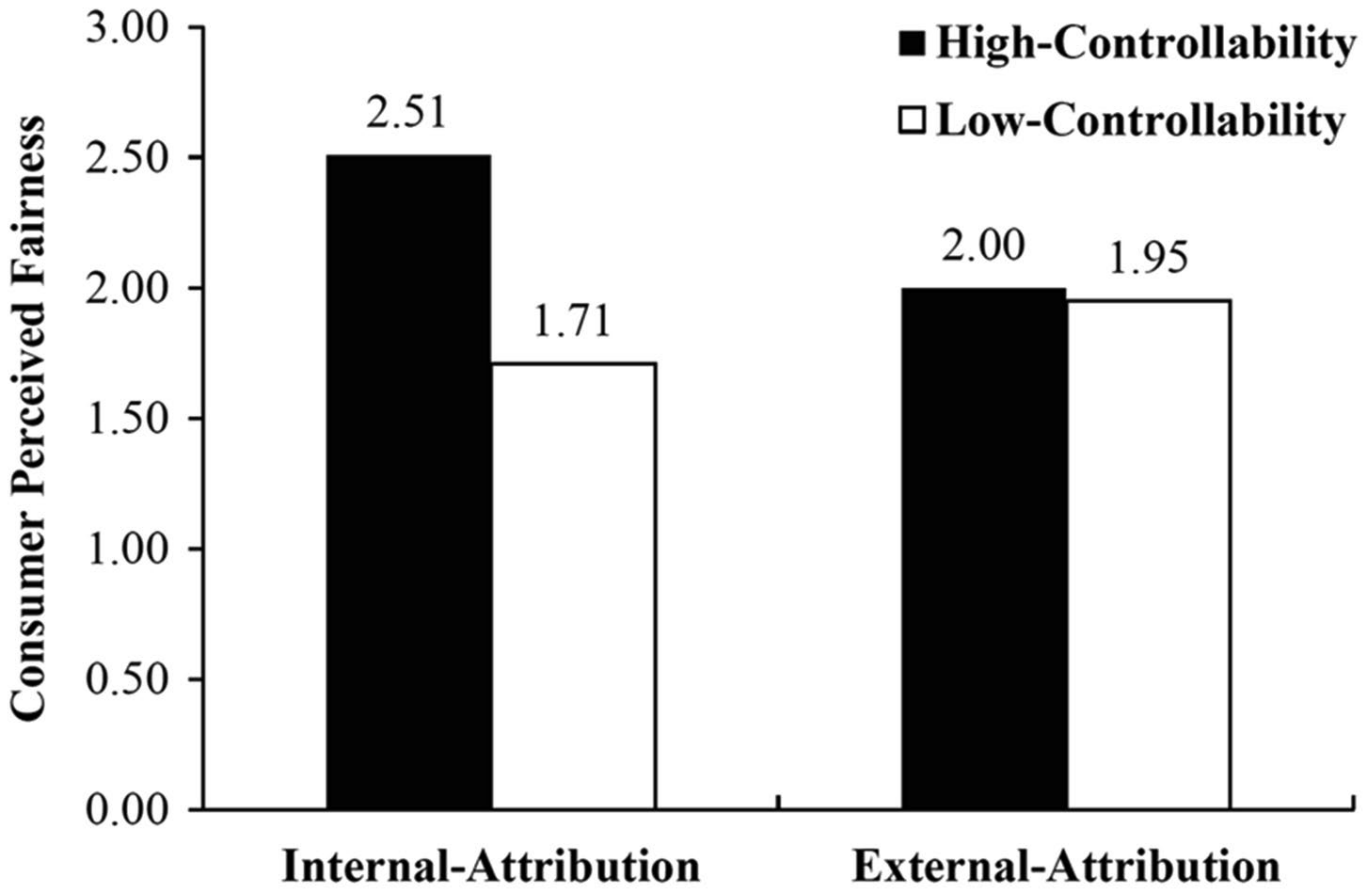
The Impact of Attribution on Perceived Fairness.

After including the interaction between controllability and locus of causality into the regression, it was found that the two-way interaction is significant (*t*(133)=2.034, *p*<0.05). As shown in Figure 2, when consumers attribute the data-driven discrimination to the firm (external attribution), the effect of controllability on perceived fairness is not significant. That is, regardless of the level of the controllability, consumer perceived fairness is relatively low (*M*_high controllability_=2.00, *M*_low controllability_=1.95, *t*(66)=0.186, *p*=0.853). When consumers attribute the data-driven discrimination to themselves (internal attribution), however, perceived fairness is significantly higher in high-controllable conditions than that in low-controllable conditions (*M*_high controllability_=2.51, *M*_low controllability_=1.71, *t*(67)=3.006, *p*<0.01). Therefore, the positive effect of controllability on perceived fairness is stronger under internal attribution (than under external attribution). Hence, H1c is supported.

#### Consumer Trust

**Figure 3:**
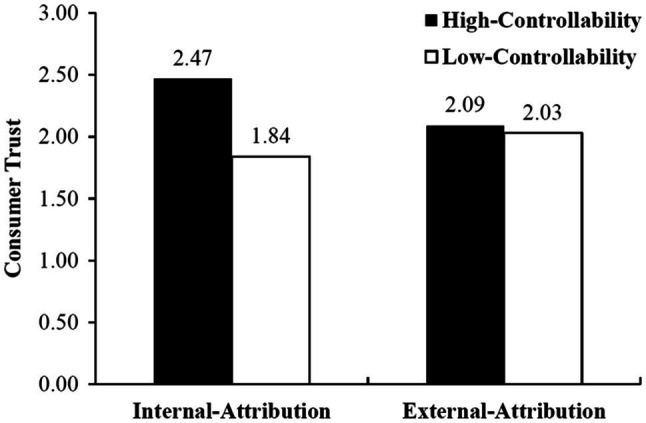
The Impact of Attribution on Consumer Trust.

Regarding consumer trust, the effect of controllability is significant and consumer trust is significantly higher in high-controllable conditions than that in low-controllable conditions (*M*_high controllability_=2.28, *M*_low controllability_=1.93, *t*(134)=2.119, *p*<0.05). Therefore, H2a is also supported. However, the effect of the locus of causality on consumer trust is not significant (*t*(134)=0.482, *p*=0.630), and thus, H2b is not supported. After including the interaction between controllability and locus of causality into the regression, it was found that the interaction effect is significant (*t*(133)=1.744, *p*<0.10). As shown in Figure 3, the effect of controllability on consumer trust is only significant when consumers attribute data-driven discrimination to themselves (internal attribution; *M*_high controllability_=2.47, *M*_low controllability_=1.84, *t*(67)=2.653, *p*<0.01). When consumers attribute data-driven discrimination to the firm (external attribution), however, the effect of controllability becomes nonsignificant (*M*_high controllability_=2.09, *M*_low controllability_=2.03, *t*(66)=0.280, *p*=0.781). Therefore, H2c is supported.

#### The Mediating Role of Perceived Fairness

In this analysis, we test whether perceived fairness mediates the significant interaction effect between controllability and the locus of causality on consumer trust. As shown above, the interaction effect between controllability and the locus of causality on perceived fairness is statistically significant (*t*(133)=2.034, *p*<0.05). Moreover, when we regress consumer trust on perceived fairness and the interaction between controllability and the locus of causality, the effect of perceived fairness is positive and significant (*t*(132)=11.034, *p*<0.001) while the interaction effect becomes nonsignificant (*t*(132)=0.455, *p*=0.65). This indicates that perceived fairness fully mediates the interaction effect between controllability and the locus of causality on consumer trust. To formally test the mediating effect of perceived fairness, we conducted a mediated moderation analysis using the process procedure in SPSS [Model 8, Hayes (2017)]. The results show that the mediated moderation is significant (95% CI=[0.0190, 0.9264], based on 5,000 samples). Therefore, H3 is supported.

Furthermore, we also explored the mediating role of consumer emotions. The results show that controllability significantly reduces consumer anger (*M*_high controllability_=3.89, *M*_low controllability_=4.18, *t*(134)=−1.809, *p*=0.073), and the chain intermediary effect “controllability – consumer perceived fairness – consumer angry – consumer trust” is significant (95%CI=[0.0008, 0.0708], based on 5,000 samples). Compared with low-controllable conditions, consumers perceive higher fairness, lower angry, and higher trust in high-controllable conditions. The locus of causality, however, mainly influences consumer guilt. Compared with external attribution, consumer guilt is significantly higher when consumers internally attribute the data-driven discrimination to themselves (*M*_internal attribution_=2.48, *M*_external attribution_=1.93, *t*(134)=2.729, *p*<0.01). Moreover, our data support a chain intermediary effect “locus of causality – guilt – consumer perceived fairness - consumer angry – consumer trust” (95%CI=[0.0007, 0.0396], based on 5,000 samples), though the total effect of locus of causality on consumer trust is not significant. These findings indicate that different emotions may play important and different roles in the impact of attribution on perceived fairness and consumer trust toward the firm, which extend the “cognitive attribution-emotion-behavior” model of Weiner (1980).

### Discussion

Study 1 shows an interactive impact of controllability and locus of causality on consumer trust and the mediating role of perceived fairness in the above relationship. The results generally support an “attribution-perceived fairness-consumer trust” model in the context of data-driven discrimination. Other than perceived fairness, however, there can be other reasons why consumers in the internal attribution and high controllability condition would have higher trust. For example, these consumers may have less privacy concerns or higher general autonomy or control perceptions, which leads them to have higher trust. To rule out these alternative explanations, we conducted another experiment using general online shoppers rather than college students as subjects. The design and scenarios are the same with those of Study 1, except that we further measured consumer privacy concerns and general perceived autonomy over personal information (Sheng et al., 2008; Robert and You, 2018). The results from this experiment have largely replicated those of Study 1. However, the interaction effect of controllability and locus of causality on either privacy concerns (*p*=0.17) or perceived autonomy (*p*=0.47) is not significant. Thus, these two alternative explanations cannot explain the interaction effect of controllability and locus of causality on consumer trust.

## Study 2: the Moderating Role of Self-Concept Clarity

In Study 2, we used another discrimination scenario to replicate the conclusions of study 1 and further examined the moderating role of self-concept clarity.

### Method

Study 2 is also a 2(controllability: high vs. low)×2(locus of causality: internal vs. external) between-subjects design. One hundred and fifty qualified undergraduate students participated in the study. All subjects were randomly assigned into one of the four conditions.

During the experiment, the subjects were first shown the description of the discrimination scenario. The subjects imagined that she/he is a loyal customer to an online retailer, which tracks customer shopping behavior and is proficient at utilizing data analytics to treat customers differently. We asked the subjects to imagine that “You and your colleague both made a purchase (of the same product) at the online retailer the other day, but the retailer only provided gifts to your colleague, who is new to the online retailer.” All subjects read the same scenario description, and the only difference for the four conditions is the attribution of the retailer’s discrimination.

For internal attribution conditions, the subjects imagined that they were treated differently because “you accidentally clicked the button of ‘Agree to Enterprise Access to Personal Data’ when registering at the retailer.” “This allows the retailer to analyze your personal data and identify that you will still be loyal to the retailer even if it does not provide you the gifts.” For external attribution conditions, the subjects imagined that they were treated differently because “the retailer (similar to other retailers) tracks and uses your behavioral data without your consent.” “This allows the retailer to analyze your personal data and identify that you will still be loyal to the retailer even if it does not provide you the gifts.” To manipulate low controllability, we asked the subjects to imagine that “you could not change the status of being discriminated at the retailer even if you change your profile settings or browsing behaviors.” In high-controllable conditions, however, the subjects imagined that “you could change the status of being discriminated at the retailer by changing your profile settings or browsing behaviors (e.g., opt out of the agreement for sharing your data with the retailer).”

After reading the scenario, we asked the subjects to report their perceived fairness of the practice of the retailer and their trust toward the retailer (5-point scale). Then, we measured the subjects’ self-concept clarity using the scale developed in previous research (Campbell et al., 1996; 5-point scale) and used a median split to classify subjects into high vs. low self-concept clarity. To be noted, we followed most prior literature to consider self-concept clarity as a stable and enduring disposition of consumers (e.g., Savary and Dhar, 2020) and did not directly manipulate self-concept clarity. The items for the manipulation check of controllability and locus of causality are similar to those of study 1. Finally, the subjects left their demographic information (e.g., gender and age) and were thanked and fully debriefed.

### Results

#### Manipulation Check

Compared to low controllability conditions, subjects in high controllability conditions perceived that the situation was significantly more controllable (*M*_high controllability_=3.52, *M*_low controllability_=2.44, *t*(148)=13.178, *p*<0.001). Furthermore, subjects in internal attribution conditions were significantly more inclined to believe that being discriminated was caused by their own reasons than those in external attribution conditions (*M*_internal attribution_=3.62, *M*_external attribution_=2.30, *t*(148)=17.850, *p*<0.001). Thus, the experimental manipulation was successful.

#### The Moderating Role of Self-Concept Clarity

We first examined the impact of attribution of data-driven discrimination on consumer trust and found similar results to those of Study 1. That is, controllability significantly improves consumer trust and the interaction between controllability and locus of causality is also significant. Then, we categorized subjects into high vs. low self-concept clarity using a median split and examined the three-way interaction of self-concept clarity, controllability, and locus of causality. The results show that the three-way interaction on consumer trust is significant (*t*(142)=−1.808, *p*<0.10; see Table 1). Moreover, the two-way interaction effect between controllability and locus of causality is also significant (*t*(142)=3.013, *p*<0.01).

**Table 1:** Regression analysis results (Study 2).

	*Coefficients*	*Std. Error*	*t-value*	*value of p*
Intercept	1.941^***^	0.198	9.817	0.000
Controllability	−0.141	0.289	−0.489	0.626
Locus of Causality	−0.341	0.269	−1.269	0.207
Self-Concept Clarity	−0.204	0.272	−0.751	0.454
Controllability × Locus of Causality	1.160^***^	0.385	3.013	0.003
Controllability × Self-Concept Clarity	0.752^*^	0.384	1.960	0.052
Locus of Causality × Self-Concept Clarity	0.215	0.380	0.567	0.571
Controllability × Locus of Causality × Self-Concept Clarity	−0.971^*^	0.537	−1.808	0.073

*** p<0.01; **p<0.05;

* *p<0.1*.

**Figure 4:**
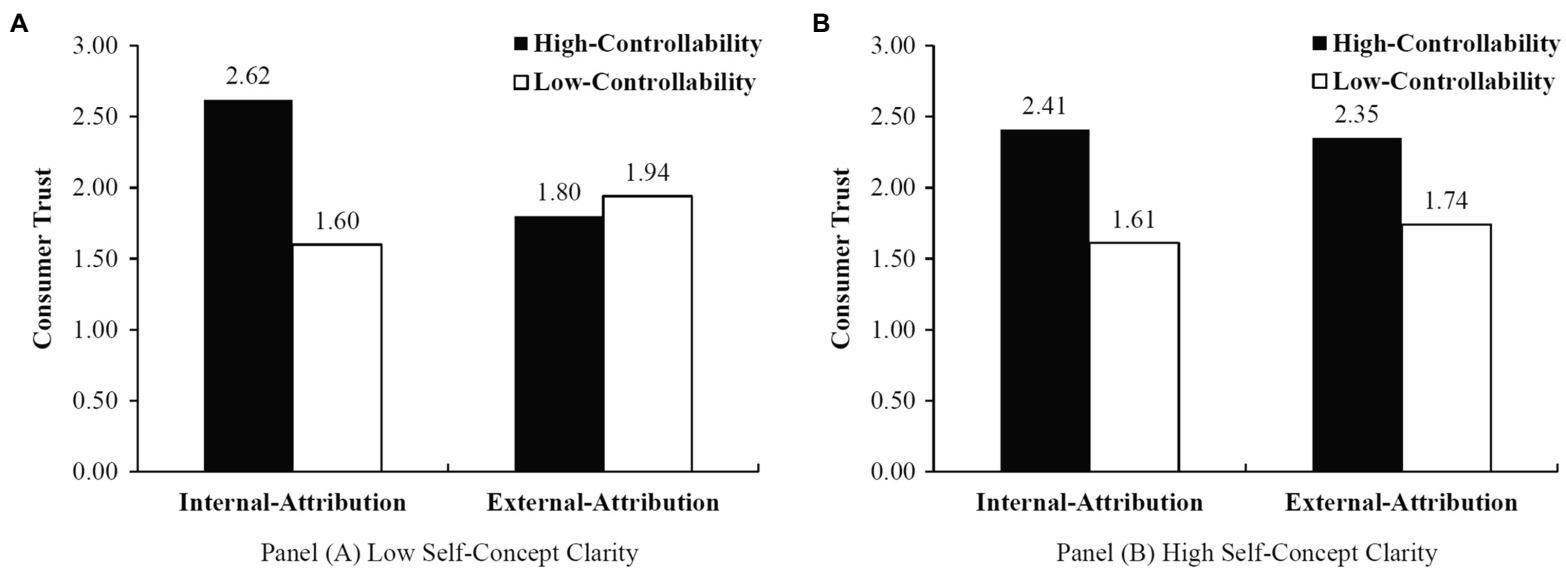
Impact of Attribution for Consumers with Low (Panel **A**) vs. High (Panel **B**) Self-Concept Clarity.

To delve into the three-way interaction and test H4a and H4b, we conducted a linear regression for subjects with high vs. low self-concept clarity separately. For subjects with low self-concept clarity, we replicated the conclusions of Study 1. As shown in Panel (A) of Figure 4, the interaction between controllability and locus of causality significantly affects consumer trust (*t*(69)=3.108, *p*<0.01). In particular, the effect of controllability on consumer trust is only significant under internal attribution (*M*_high controllability_=2.62, *M*_low controllability_=1.60, *t*(39)=3.866, *p*<0.001). For external attribution, regardless of controllability, consumer trust is relatively low (*M*_high controllability_=1.80, *M*_low controllability_=1.94, *t*(30)=−0.557, *p*=0.581). As a result, H4a is supported. For subjects with a clear and accurate self-concept, however, the interaction between controllability and locus of causality is not significant (*t*(73)=0.493, *p*=0.623). For these subjects, as shown in Panel (B) of Figure 4, no matter whether being discriminated is caused by themselves or by the firm, consumer trust in low-controllable conditions is significantly lower than that in high-controllable conditions (internal attribution: *M*_high controllability_=2.41, *M*_low controllability_=1.61, *t*(33)=2.874, *p*<0.01; external attribution: *M*_high controllability_=2.35, *M*_low controllability_=1.74, *t*(40)=2.319, *p*<0.05). Thus, H4b is also supported.

### Discussion

Study 2 used another discrimination scenario and replicated the findings of Study 1. More importantly, Study 2 examines a boundary condition of the interaction effect of controllability and locus of causality on consumer trust, in which the interaction effect holds solely for consumers with low self-concept clarity. For consumers with high self-concept clarity, only the main effect of controllability on consumer trust is significant. This may be because consumers with high self-concept clarity are more problem-solving-oriented and care more about how to avoid being discriminated by strategically changing their behaviors, rendering them less likely to be affected by the locus of causality and more likely to be influenced by controllability.

To be noted, similar to the results of Study 1, Study 2 shows no significant impact of locus of causality on perceived fairness or trust. We conjecture that the nonsignificant impact of locus of causality in our research setting can be caused by a ceiling effect. Data-driven discrimination, especially discrimination against the firm’s loyal customers, breaks social norms and cannot be easily justified or rationalized by the firm. Even if the firm claims that consumers themselves (rather than the firm) can be responsible for their being discriminated, consumers may still feel highly unfair (and have lower trust) when facing such discrimination. In this circumstance, locus of causality (internal or external) may not significantly affect perceived fairness and consumer trust.

## General Discussion

This research employs attribution theory to investigate how consumers respond to a new type of discrimination that emerges with the development of big data analytics – data-driven discrimination. We conducted two laboratory experiments to examine how the different attributions of data-driven discrimination from a consumer perspective (i.e., controllability and locus of causality) affect perceived fairness and consumer trust toward the firm and obtained some interesting findings.

First, we show a significant interaction effect between the controllability and locus of causality of data-driven discrimination on consumer perceived fairness. The controllability by consumers can only increase consumer perceived fairness when consumers attribute the discrimination internally to themselves. The effect of locus of causality, however, cannot significantly affect perceived fairness. Thus, consumers generally perceive data-driven discrimination to be highly unfair even if being discriminated may be due to their own reasons. This finding is inconsistent with previous research which suggests that consumers perceive a price increase to be more unfair if they attribute the price increase internally to the firm rather than external factors (e.g., Xia et al., 2004). The nonsignificant impact of locus of causality in our research setting could be caused by a ceiling effect, in which data-driven discrimination (with either internal or external attribution) generates extremely unfair perceptions. Second, we show that the above interaction effect on perceived fairness can be transferred to consumer trust. This finding indicates an “attribution-perceived fairness-consumer trust” model, and alternative mechanisms through privacy concerns or perceived autonomy over personal information are not supported. Hence, data-driven discrimination can greatly erode consumer trust toward the firm, especially when the discrimination is attributed in certain ways (e.g., external attribution or internal attribution with low controllability) and high unfairness perceptions are generated. This suggests that data-driven discrimination may have profound influence on a firm’s customer relationship management. Finally, the interaction effect between the controllability and locus of causality on consumer trust varies according to consumer self-concept clarity. The interaction effect holds only for consumers with low self-concept clarity; for consumers with high self-concept clarity, controllability plays a dominant role.

Theoretically, this paper contributes to related literature on consumer response to discrimination and attribution theory. Despite the widespread attention aroused by data-driven discrimination, little empirical research examines how consumers respond to it. Previous research on consumer response to price discrimination only examines its general negative impact on perceived fairness and consumer trust, whether and when consumers respond to discrimination less negatively, especially when the discrimination is data-driven and norm-breaking, remains understudied. This article fills the research gap and provides insights into how the attribution of data-driven discrimination may affect consumer fairness perception and trust toward the firm. Second, we extend attribution theory to examine consumer response to a new type of discrimination emerging in data-rich environments. Different from previous research, we innovatively discuss the attributions of data-driven discrimination (i.e., controllability and locus of causality) from the consumer perspective and examine how these two interactively influence consumer response. These investigations are new to the literature and provide additional insights (beyond those in previous literature) into how attributions may affect consumer fairness perceptions. For example, in our research setting, we show that controllability and locus of causality have an interaction effect on consumer response to data-driven discrimination, while locus of causality *per se* has no significant effect. Finally, based on the “cognitive attribution-emotion-behavior” model, the results of this paper suggest an “attribution-perceived fairness-consumer trust” model for consumer response to data-driven discrimination. Our results also reveal the important role that different emotions play in the relationship between attribution and consumer trust. Our conclusions illuminate the underlying mechanism and boundary condition of the interaction effect between controllability and locus of causality regarding data-driven discrimination on consumer trust, thereby providing fine-grained insights into how and when attribution may reshape consumer trust.

The conclusions of this article also provide some managerial implications. First, when strategically deciding whether to involve in data-driven discrimination, firms may survey possible consumer attributions of their data-driven discrimination and take these attributions into serious consideration. Increasingly more firms are involving in the practice of data-driven discrimination, but few of them are aware of how certain attributions can generate extremely unfair perceptions and ruin their reputation among consumers. Second, when firms encounter a crisis due to discriminating consumers using data analytics, they had better emphasize the potential ways through which consumers can avoid being discriminated (i.e., the controllability by consumers). This is because controllability by consumers may help firms attenuate the negative effects of discrimination (e.g., eroded trust). Furthermore, the firm should also pay attention to consumers’ attribution of locus of causality of its data-driven discrimination as whether communicating controllability with consumers is effective further depends on locus of causality. When consumers externally attribute the discrimination to the firm or other parties, for instance, consumer perceived fairness and trust toward the firm can be very low even if consumers have volitional control over whether to be discriminated. In the face of this situation, the firm may need to apologize publicly and make substantial compensation to consumers in a timely manner, so as not to greatly erode consumer trust. Third, when firms are dealing with crises caused by its practice of data-driven discrimination, they may also need to adjust their strategies according to consumer characteristics such as self-concept clarity. For consumers with high self-concept clarity, communicating controllability can be an effective strategy to maintain consumer trust. For consumers with low self-concept clarity, however, communicating controllability only works for consumers who attribute the discrimination internally to themselves. Finally, when implementing data-driven discrimination, firms should be especially cautious of perceived fairness and consumer emotions, as they mediate the effect of the attribution of data-driven discrimination on consumer trust. Firms may try to introduce its practice of data-driven discrimination to consumers in more justified ways, which helps firms increase consumer perceived fairness and consumer trust. Firms should also be wary of consumers’ negative emotional reactions to their data-driven discrimination, as emotions like anger may be the key between unfairness perception and eroded trust. Consumer guilt (aroused by internal vs. external attribution), however, can generate higher fairness perception, lower anger, and higher trust. Thus, when facing a crisis, inducing consumers to be guilty may help firms attenuate the negative effects of involving in data-driven discrimination.

As the first attempt to examine consumer response to data-driven discrimination, this article has several limitations awaiting further research. First, we mainly focus on data-driven discrimination with loyalty penalty, in which the discrimination is against the firm’s loyal customers. Future research may extend our research to examine consumer response to more general forms of data-driven discrimination where no loyalty penalty is involved. Second, in Study 2, we measured (rather than manipulated) consumer self-concept clarity and classified subjects into high vs. low self-concept clarity by a median split. Though this is common practice in consumer behavior literature, this may to some extent compromise our internal validity. Therefore, future research may directly manipulate consumer self-concept clarity and more formally examine its moderating role. Third, we show the effect of attribution regarding data-driven discrimination on consumer trust using only two studies with two specific discrimination scenarios. As data-driven discrimination has diverse forms, future research may replicate our findings using other more interesting discrimination scenarios. Finally, similar to most previous research, we used laboratory experiments to ensure internal validity and establish causal relationship. Future research can examine the effect of attribution of data-driven discrimination in more realistic settings and use more general population as subjects, to enhance the external validity.

## Data Availability Statement

The raw data supporting the conclusions of this article will be made available by the authors, without undue reservation.

## Ethics Statement

Ethical review and approval was not required for the study on human participants in accordance with the local legislation and institutional requirements. The patients/participants provided their written informed consent to participate in this study.

## Author Contributions

LS and YT: conceived and designed the study, literature search, and synthesis. YT: data collection and analysis. LS: wrote the paper. All authors contributed to the article and approved the submitted version.

## Funding

This research was supported by the National Natural Science Foundation of China (Grants 71972195, 71502182, and 71702208).

## Conflict of Interest

The authors declare that the research was conducted in the absence of any commercial or financial relationships that could be construed as a potential conflict of interest.

## Publisher’s Note

All claims expressed in this article are solely those of the authors and do not necessarily represent those of their affiliated organizations, or those of the publisher, the editors and the reviewers. Any product that may be evaluated in this article, or claim that may be made by its manufacturer, is not guaranteed or endorsed by the publisher.
